# Erectile dysfunction and exposure to ambient Air pollution in a nationally representative cohort of older Men

**DOI:** 10.1186/s12940-017-0216-6

**Published:** 2017-02-17

**Authors:** Lindsay A. Tallon, Justin Manjourides, Vivian C. Pun, Murray A. Mittleman, Marianthi-Anna Kioumourtzoglou, Brent Coull, Helen Suh

**Affiliations:** 1 Department of Health Sciences, Northeastern University, 360 Huntington Avenue, Boston, MA 02115 USA; 2 MCPHS University, 179 Longwood Avenue, Boston, MA 02115 USA; 3 Department of Epidemiology, Harvard T.H. Chan School of Public Health, 677 Huntington Ave., Boston, MA 02115 USA; 4 Department of Environmental Health Sciences, Columbia University Mailman School of Public Health, 722 W. 168th Street, #1105C, New York, NY 10032 USA; 5 Department of Biostatistics, Harvard T.H. Chan School of Public Health, 677 Huntington Ave., Boston, MA 02115 USA; 6 Department of Civil and Environmental Engineering, Tufts University, 200 College Avenue, 301 Anderson Hall, Medford, MA 02155 USA

**Keywords:** Men, Vasculature, Erectile dysfunction, PM_2.5_

## Abstract

**Background:**

Little is known about the association between air pollution and erectile dysfunction (ED), a disorder occurring in 64% of men over the age of 70, and to date, no studies have been published. To address this significant knowledge gap, we explored the relationship between ED and air pollution in a group of older men who were part of the National Social Life, Health, and Aging Project (NSHAP), a nationally representative cohort study of older Americans.

**Methods:**

We obtained incident ED status and participant data for 412 men (age 57–85). Fine particulate matter (PM_2.5_) exposures were estimated using spatio-temporal models based on participants’ geocoded addresses, while nitrogen dioxide (NO_2_) and ozone (O_3_) concentrations were estimated using nearest measurements from the Environmental Protection Agency’s Air Quality System. The association between air pollution and incident ED (newly developed in Wave 2) was examined and logistic regression models were run with adjusted models controlling for race, education, season, smoking, obesity, diabetes, depression, and median household income of census tract.

**Results:**

We found positive, although statistically insignificant, associations between PM_2.5_, NO_2_, and O_3_ exposures and odds of incident ED for each of our examined exposure windows, including 1 to 7 year moving averages. Odds ratios (OR) for 1 and 7 year moving averages equaled 1.16 (95% CI: 0.87, 1.55) and 1.16 (95% CI: 0.92, 1.46), respectively, for an IQR increase in PM_2.5_ exposures. Observed associations were robust to model specifications and were not significantly modified by any of the examined risk factors for ED.

**Conclusions:**

We found associations between PM_2.5_, NO_2_, and O_3_ exposures and odds of developing ED that did not reach nominal statistical significance, although exposures to each pollutant were consistently associated with higher odds of developing ED. While more research is needed, our findings suggest a relationship between air pollutant exposure and incident cases of ED, a common condition in older men.

**Electronic supplementary material:**

The online version of this article (doi:10.1186/s12940-017-0216-6) contains supplementary material, which is available to authorized users.

## Background

Erectile dysfunction (ED), defined as the inability of a man to achieve or maintain an erection [1], is one of the most common health conditions affecting older men. Prevalence of ED is estimated to equal 20–40% in men aged 60–69 years and 64% in men over the age of 70 [2], with 617,715 new cases of ED expected each year in the United States among white males between 40 and 69 years old [3]. With the aging of the population, it is estimated that globally by the year 2025, over 320 million men will have ED [4]. ED has a considerable impact on quality of life, having been associated with a variety of underlying psychological and physiological conditions, such as depression, Type 1 and Type 2 diabetes mellitus, use of medications, and cardiovascular disease [5]. However, ED is often left untreated, with as few as 10% of men with ED receiving treatment [6]. Both treatment and prevention of ED includes lifestyle modifications like exercise, weight loss, smoking cessation, and treatment for comorbid conditions [5].

ED is a complex neurovascular process, influenced by hormones and involves multiple pathways within the body. ED is a predictor of occult and subclinical cardiovascular disease [7] that often occurs in 3 to 5 years after ED diagnosis [7, 8]. Given this, it is not surprising that ED has been shown to share a number of risk factors with cardiovascular disease, including restricted blood flow and vascular compromise with endothelial dysfunction at both the macro and microvascular level [9, 10]. It is also plausible that ED shares other risk factors with cardiovascular disease, including air pollutants such as fine particulate matter (PM_2.5_; particles with aerodynamic diameters ≤2.5 μm) [11]. PM_2.5_ in particular has consistently been shown to be strongly associated with cardiovascular morbidity and mortality [12, 13]. The biological pathways through which PM_2.5_ leads to cardiovascular damage is thought to occur primarily through inflammation and oxidative stress [14], leading to systemic vascular and endothelial cell dysfunction [14, 15]. Whether PM_2.5_’s impacts on systemic vascular dysfunction also lead to ED is unknown. To our knowledge, no studies to date have been published investigating the association between air pollution and ED. To address this significant knowledge gap, we investigated the association between air pollution and incident ED using a nationally representative cohort study of older men from the National Social Life, Health, and Aging Project (NSHAP).

## Methods

### Study participants

NSHAP is a longitudinal, population-based study of community-dwelling older adults, who were selected for participation based on a nationally representative probability sample of household-resident older adults aged 57–85 years, with oversampling of African-Americans, Hispanics, men, and adults aged 75–84 years. Numerous self-reported and biological measures of social, psychological, functional, and physiological health were collected for each participant in each of two data collection waves. The first wave (Wave 1) was conducted from July 2005 to March 2006, with in-home interviews, bio-specimen collection, and respondent-completed questionnaires performed for 3,005 individuals (1,455 men). The same data were obtained in Wave 2 (August 2010 to May 2011) for 3,377 participants (1,538 men), including 2,261 respondents from Wave 1, 161 Wave 1 eligible, but non-interviewed respondents, and 955 spouses or cohabitating romantic partners. The overall weighted response rate (weighted for differential probabilities of selection and nonresponse) among the 2,261 individuals (1068 men) who participated in both waves was 75.5% and 76.9% for Waves 1 and 2 respectively, and interviews were completed in English or Spanish as appropriate.[16] The protocol was approved by the Institutional Review Boards of Northeastern University, the University of Chicago and NORC at the University of Chicago. All respondents provided written informed consent.

### ED measures

ED status was obtained through self-report to the question asking men if “during the last 12 months has there ever been a period of several months or more when you had trouble getting or maintaining an erection?” This question was administered only to men who reported sexual activity within one year of the interview date in Wave 1 and of all men in Wave 2. Information on ED medication use and prostatectomy surgery was also collected via questionnaire. Men were counted as having ED if they answered yes to having ED or taking medications to treat ED. Incident cases were defined as men who participated in both waves and developed ED between Wave 1 and Wave 2.

### Other covariates

Measures of height and weight were taken at the time of the interview to calculate to body mass index (BMI), with a BMI of 30 or greater kg/m^2^ defined as obese [17]. Additionally, blood spot samples were collected and blood pressure was measured for each participant (2–3 consecutive times) during the home interview. Blood pressure was estimated as the average of the repeated measurements, with hypertension defined as having average systolic and diastolic measurement of greater than 140 mmHg and 90 mmHg, respectively [18]. C-Reactive Protein (CRP) was measured from dried blood samples, and was considered elevated if greater than 1 mg/L. Participants with CRP measurements greater than 40 mg/L were not included in CRP analyses, given the likelihood of an active infection [19].

In addition, information on medical conditions, like diabetes, and measures of activity, such as exercise and ability to walk across a room, were obtained through participant self-report. Measures of emotional health were also obtained, with depression assessed using an eleven question version of the Center for Epidemiological Studies – Depression scale, CESD-11 [20]. A score of 9 or greater on the CESD-11 was used to identify individuals with “moderate-to-severe” depressive symptoms [20, 21].

Finally, information on census tract characteristics including the tract median household income was obtained from the 2010 US Census and matched to participants based on their geocoded residence. A participant’s place of residence was also used to determine the geographic region of the United States in which they resided (West, Midwest, Central, South, Northeast). The regional divisions are based off of the Environmental Protection Agency’s (EPA’s) regional planning organizations.

### Air pollution exposure assessment

Daily PM_2.5_ exposures were estimated on a 6 km (km) grid covering the conterminous United States using a set of five spatio-temporal generalized additive mixed models (GAMMs) of daily PM_2.5_ mass levels fit separately to 1999–2001, 2002–2004, 2005–2007, 2008–2009, and 2010–2011 based on previous work from Yanosky et al. [22]. PM_2.5_ data for the models were obtained primarily from the EPA’s Air Quality System (AQS) database and Interagency Monitoring of Protected Visual Environments (IMPROVE) network [23, 24]. The models included three meteorological covariates (i.e., wind speed, temperature, and total precipitation) that influence pollutant dispersion as well as several geospatial covariates such as smoothed county population density from the 2000 U.S. census, point-source PM_2.5_ emissions density within 7.5 km, proportion of urban land use within 1 km, elevation, and annual-average PM_2.5_ for 2002 from EPA’s Community Multiscale Air Quality model. Finally, the daily PM_2.5_ models include traffic-related PM levels, represented as the output of a Gaussian line-source dispersion modeling approach. The line-source model uses ADMS-Roads software and associated spatially-smoothed traffic intensity and daily meteorological inputs to describe small-scale spatial gradients in primary PM concentrations near roadways. The daily PM_2.5_ model was validated using cross-validation techniques, as in Yanosky et al. [22], and had a cross-validation R^2^ of 0.76. Cases and controls were matched by date of interview to the PM_2.5_ grid values closest to their residential addresses, taking into account moves between waves. One to seven year moving averages from the date of interview were calculated from the daily PM_2.5_ estimates. The mean distance between each grid centroid–residential address pair was 2.23 km, with a range of 0.05 – 4.21 km.

As secondary analyses, exposures to ozone (O_3_) and nitrogen dioxide (NO_2_) were also estimated using data from the EPA’s AQS. For both O_3_ and NO_2_, participants were matched to the AQS monitor nearest to their residential address, provided that this monitor was located within 60 km of the participant’s home. Air pollution exposures were estimated for 93% (for O_3_) and 63% (for NO_2_) of all male NSHAP participants. For NO_2_, 1–7 year moving averages from the date of interview were calculated. For O_3_, 1–7 year average exposures preceding the interview date were calculated based on the average warm season (April-September) concentration, since O_3_ is measured at most monitoring sites only during warmer months. Moving averages for each pollutant were considered valid if ≥75% of the daily values within each exposure window were available.

### Statistical analyses

To investigate the association between chronic air pollutant exposures and incident ED, we performed logistic regression analyses, with the outcome of incident ED defined as men reporting either ED or ED medication use in Wave 2 but not Wave 1. Base models controlled for covariates including age and geographic region, while adjusted models additionally controlled for race/ethnicity (white, black, Hispanic non-black, other), education (proxy variable for socioeconomic status (SES), less than high school, completed high school or vocational school, college degree), current smoking status, obesity, diabetes, depression, season, and median household income of the census tract. Covariates were included based upon previous, known associations with either air pollution exposures or ED. Results for all models are expressed as the odds ratios (ORs) of having ED per interquartile range (IQR) increase in ambient air pollution exposure.

Due to missingness in ED, BMI, and diabetes (39.6% missing incident ED outcome in Wave 1 and 13.6% outcome missing in Wave 2, 5.1% missing BMI in both waves, and 0.6% missingness for diabetes in both waves), we used multiple imputation to impute missing data. We did so using the fully conditional specification (FCS) algorithm with 100 imputed datasets created given the logistic model and binary data used in the model [25]. Variables used in imputation included the outcome of incident ED, any factors relating to ED (CRP, ability to walk across a room, antiandrogens, and beta blockers) as well as all covariates included in the model. Imputation resulted in a data set containing all men who participated in both waves (*n* = 1068).

We examined effect modification of the association between PM_2.5_ and incident ED by including separate interactions between PM_2.5_ and obesity, hypertension, diabetes, CRP, exercise (as less or greater than once each week), current smoking, and depression. These potential modifiers were selected based on previous scientific findings for air pollution and cardiovascular disease. All effect modification analyses were performed for 4 year moving average PM_2.5_ exposures since it was the interval with the strongest observed effect estimate.

Sensitivity analyses were performed to examine the robustness of our findings to (1) alternate definitions of ED and geographic region, (2) residential moves, (3) treating ED as a reversible outcome, and (4) imputation of ED data. We examined the robustness of our findings with regard to our definition of ED as well as how the geographic regions of the U.S. were defined. We did so repeating our primary analyses but defining ED as only those men who reported having ED (*n* = 129) as opposed to also including men reporting ED medication usage and by defining region based on 4 (North, South, East, and West) as opposed to the 5 regions used in the primary analyses. We further assessed the sensitivity of our findings to residential moves by restricting the analyses to men who did not move during the study. Finally, to explore the relation of air pollution to ED prevalence while treating ED as a reversible outcome [26], we included all cases of ED (*n* = 746) in generalized mixed models with random intercepts, rather than just incident cases of ED. As before, models included adjustments for age, race/ethnicity, education, year, season, current smoking, obesity, diabetes, depression, median household income, and region. Similar to our primary analyses, additional covariates were selected based on previous scientific findings to investigate effect modification. Finally, complete case analyses were undertaken to examine the potential effects of imputation.

All statistical analyses were conducted using SAS version 9.4 software (SAS Institute Inc., Cary, North Carolina). Statistical significance is reported at the two-sided 0.05 level.

## Results

In Wave 2, 132 men answered yes for the first time to either having ED (129 men) or to ED medication use (10 men total, 3 men with medication usage alone), with these men defined as incident ED cases for our study period (Table 1). The number of incident ED cases was small relative to the number of men reporting ED in Wave 1, for which 331 men reported having ED and 29 reported being on ED medication. Over both waves, 280 men did not report developing ED and were the comparison group in our main analysis (Table 1). The majority of men with incident ED were non-Hispanic White (78.79%), with the average age of 70.3 years. Compared to men without ED, those with ED were more likely to be non-Hispanic Whites, have higher education levels, and be obese. Other demographic characteristics of men with and without ED were similar. One year mean moving averages for PM_2.5_ during Wave 2 were 8.9 μg/m^3^ (±2.1) for men with ED and 8.6 μg/m^3^ (±2.3) for men without ED. A comparison of men with and without data for prevalent ED (provided in Additional file 1: Table S1) showed that, depending on the wave, men differed significantly in the distribution of major risk factors for ED including age, education, diabetes status, obesity rates, depression and stress.

**Table 1:** Characteristics of NSHAP Male Participants Included in the Incident ED Study^a^

Characteristic	Men with ED	Men without ED	*P*-value^b^
Number of Participants^c^	132	280	
Age, mean (SD)	70.73 (6.27)	69.55 (6.06)	0.07
Race, n (%)			
Non-Hispanic white	104 (78.79%)	186 (66.67%)	0.01
Non-Hispanic black	17 (12.88%)	39 (13.98%)	
Hispanic non-black	8 (6.06%)	43 (15.41%)	
Other	3 (2.27%)	11 (3.94%)	
Region			
Northeast	24 (18.18)	56 (20.07)	0.03
Southeast	37 (28.03)	54 (19.35)	
Midwest	26 (19.70)	39 (13.98)	
Central	18 (13.64)	47 (16.85)	
West	27 (20.45)	83 (29.75)	
Education Level, n (%)			
Less than High School	11 (8.33%)	60 (21.43%)	0.004
H.S. or vocational school	74 (56.06%)	131 (46.79%)	
College degree or greater	47 (35.61%)	89 (31.79%)	
Median Household Income^d^, mean (SD)	58.97 (24.54)	61.09 (31.01)	0.49
Diabetes, n (%)	37 (28.42%)	44 (15.71%)	0.003
Hypertension^e^, n (%)	53 (40.46%)	120 (43.80%)	0.52
Elevated CRP, (>1), n (%)	86 (65.65%)	161 (57.91%)	0.14
BMI (kg/m^2^), mean (SD)	30.16 (5.66)	29.09 (5.37)	0.07
Exercise (weekly or more), n (%)	91 (68.94%)	197 (70.36%)	0.77
Current smoker, n (%)	17 (12.88%)	46 (16.43%)	0.35
Elevated depression^f^, n (%)	23 (17.42%)	39 (13.93%)	0.35
PM_2.5_^g^ (μg/m^3^), mean (SD)	8.87 (2.12)	8.63 (2.30)	0.32

^a^Statistics are given for male participants from ED analysis, results are given as n (%) or mean (SD)

^b^ *p* values calculated for difference between groups using chi-square tests for categorical variables and *t*-test for difference in means

^c^Men with ED are defined as subjects replying yes to ED question (129) or reporting ED medication usage (10) in Wave 2 and not Wave 1 (7 men replied yes to both); Men without ED are subjects who replied no to ED question and ED medication use in both waves

^d^Median household income is from census tract of each participant, results given in thousands of dollars

^e^>90 mmHg diastolic and/or >140 mmHg systolic averaged from 2 or 3 blood pressure measurements

^f^Elevated depression defined as a score of 9 or greater on the CESD-11

^g^PM_2.5_ = Particulate matter ≤ 2.5 μm in diameter, 1 year moving average values estimated using concentration estimated at closest 6x6km grid point to participant residence using GIS-based spatio-temporal models

Table 2 presents the associations between incident ED and moving average PM_2.5_ exposures over 1 to 7 years prior to the interview date for both base and adjusted models for imputed data. An IQR increase in 1 year moving average PM_2.5_ exposure (3.25 μg/m^3^) was associated with a 1.16 (95% CI: 0.87, 1.55) times higher odds of developing ED, with similar results for an IQR increase in the 7 year moving average exposure (3.10 μg/m^3^, OR = 1.16 (95% CI, 0.92, 1.46)). The association between PM_2.5_ and ED was not significantly modified by adverse health conditions, such as being obese or having elevated CRP, diabetes, hypertension, depression, or by behaviors such as current smoking or exercise (Table 3), although the estimated ORs were consistently higher for individuals without adverse health conditions.

**Table 2:** ORs (95% CIs) for Incident ED per IQR Increase in PM_2.5_^a^

	PM_2.5_ Moving Average^b^						
	1 year	2 year	3 year	4 year	5 year	6 year	7 year
Base Model^c^	1.08 (0.84, 1.40)	1.07 (0.84, 1.36)	1.08 (0.85, 1.35)	1.09 (0.87, 1.36)	1.08 (0.88, 1.34)	1.08 (0.88, 1.34)	1.08 (0.88, 1.32)
Adjusted Model^d^	1.16 (0.87, 1.55)	1.16 (0.89, 1.53)	1.17 (0.90, 1.52)	1.17 (0.91, 1.50)	1.16 (0.91, 1.48)	1.16 (0.92, 1.47)	1.16 (0.92, 1.46)

^a^Incident ED is defined as either replying yes to ED question or reported ED medication usage in Wave 2 but not in Wave 1, multiple imputation using FCS algorithm run 100 times, results given are mean ORs (95% Confidence Intervals)

^b^PM_2.5_ 1 year IQR = 3.25 μg/m^3^, 2 years IQR =3.12 μg/m^3^, 3 years IQR = 3.18 μg/m^3^, 4 years IQR = 3.12 μg/m^3^, 5 years IQR =3.10 μg/m^3^, 6 years IQR = 3.15 μg/m^3^, 7 years IQR = 3.10 μg/m^3^

^c^Logistic regression models include adjustment for age and geographic region (West, Midwest, Central, South, Northeast) in base models

^d^Additional adjustment for ethnic group, education (<H.S., H.S., College), current smoking status, obesity, depression, diabetes, season, and median household income in adjusted models

**Table 3:** Effect Modification of the 4 Year Moving Average PM_2.5_-Incident ED Association^a^

Effect Modifier	Odds Ratio (95% CI)
Obesity	
BMI <30 kg/m^2^	1.14 (0.88, 1.46)
BMI ≥30 kg/m^2^	0.92 (0.74, 1.14)
CRP	
Low (<1 mg/L)	1.19 (0.92, 1.52)
Elevated (≥1 mg/L)	0.92 (0.75, 1.14)
Diabetes	
No	1.13 (0.86, 1.48)
Yes	0.94 (0.74, 1.19)
Hypertension	
No	1.16 (0.91, 1.48)
Yes	1.03 (0.85, 1.24)
Depression	
No	1.13 (0.84, 1.52)
Yes	0.95 (0.73, 1.23)
Exercise	
None	1.16 (0.90, 1.49)
≥1 times/week	1.03 (0.83, 1.27)
Current smoker	
No	1.19 (0.89, 1.61)
Yes	1.04 (0.80, 1.35)

^a^Results for Wave 2 with imputed data; Incident ED is defined as men who either replied yes to ED question or reported ED medication usage in Wave 2; the comparison group of men without ED replied no to the ED question and ED medication use in both waves; logistic model includes adjustment for age, geographic region, ethnic group, education (<H.S., H.S., College), current smoking status, obesity, diabetes, depression, season, median household income; Reference group is PM_2.5_ 4 year moving average exposures (IQR =3.12 μg/m^3^)

As for PM_2.5_ exposures, 1–7 year moving averages of O_3_ and NO_2_ exposures were positively associated with odds of having incident ED, although associations did not reach nominal statistical significance (Table 4). ORs increased with longer moving averages, with the largest odds ratio for O_3_ and incident ED observed for an IQR increase in exposures averaged over 2 years (OR = 1.29, 95% CI, 0.93, 1.79).

**Table 4:** ORs (95% CIs) for Incident ED per IQR Increment of O_3_ and NO_2_^a^

	Moving Averages						
	1 year	2 year	3 year	4 year	5 year	6 year	7 year
O_3_^b^ (*n* = 375)	1.27 (0.89, 1.82)	1.29 (0.93, 1.79)	1.19 (0.86, 1.64)	1.20 (0.88, 1.65)	1.17 (0.87, 1.57)	1.24 (0.92, 1.68)	1.24 (0.92, 1.66)
NO_2_^c^ (*n* = 265)	1.10 (0.70, 1.71)	1.01 (0.64, 1.58)	1.07 (0.70, 1.63)	1.11 (0.70, 1.76)	1.10 (0.71, 1.72)	1.12 (0.71, 1.76)	1.02 (0.66, 1.59)

^a^Incident ED is defined as either replying yes to ED question or reporting ED medication use in Wave 2 but not in Wave 1; 125 men had incident ED with O_3_ data available and 84 men had incident ED with NO_2_ data available; results given are ORs (95% Confidence Intervals); Logistic regression models include adjustment for age, geographic region (West, Midwest, Central, South, Northeast), ethnic group, education (<H.S., H.S., College), current smoking status, obesity (BMI ≥30), diabetes, depression, season, and median household income

^b^Summer (April-Sept) only, 1 year IQR = 8.21 ppb, 2 years IQR = 7.17 ppb, 3 years IQR = 7.13 ppb, 4 years IQR = 7.16 ppb, 5 years IQR = 6.77 ppb, 6 years IQR = 6.96 ppb, 7 years IQR = 6.81 ppb

^c^1 year IQR = 7.49 ppb, 2 years IQR = 7.35 ppb, 3 years IQR =7.18 ppb, 4 years IQR = 7.82 ppb, 5 years IQR = 7.57 ppb, 6 years IQR = 7.80 ppb, 7 years IQR = 7.81 ppb

Analyses conducted using different definitions of ED and geographic regions as well as restricting analyses to men who did not have residential moves between waves showed qualitatively comparable results. For example, including only those men who answered yes to the ED question as men with incident ED (*n* = 129), in adjusted models we found PM_2.5_-associated ORs ranging from 1.10 (95% CI, 0.75, 1.62) for an IQR increase in the 1 year moving average exposure to 1.12 (95% CI, 0.82, 1.52) for an IQR increase in the 7 year moving average exposure. Analyses of all ED cases from both waves, assuming ED is a reversible outcome, showed no statistically significant association with pollutant exposures. For example, the OR associated with IQR increases in PM_2.5_ 7 year moving average exposures was 1.06 (95% CI, 0.86, 1.30, Additional file 1: Table S2).

Analyses restricted to complete cases (Additional File 1: Table S3), had similar results to imputed data (Table 2) but with slightly lower estimates. An IQR increase in 1 year moving average PM_2.5_ exposure was associated with a 1.11 (95% CI: 0.76, 1.61) times higher odds of developing ED, while an IQR increase in 7 year moving average PM_2.5_ exposure was associated with a 1.14 (95% CI: 0.84, 1.54) times higher odds of developing ED.

## Discussion

In our nationally representative study of older men, we found that the odds of developing ED were higher but did not reach nominal statistical significance for IQR increases in PM_2.5_ exposures, with the 4 year moving average exposure having the highest odds ratio (1.17, 95% CI: 0.91, 1.50). The observed associations between recent long-term average PM_2.5_ level and ED were not significantly modified by any of the examined risk factors for ED.

This study is the first to investigate the association between incident ED and exposure to ambient air pollution. Although not statistically significant, we found PM_2.5_ exposures to be consistently associated with higher odds of developing ED. Our findings were consistent across a broad range of sensitivity analyses. The relatively small study size may in part account for the imprecise estimates of association, as we had limited power to detect associations in the range of the observed estimates.

Support for the hypothesis that ambient air pollution may impact ED incidence is provided by epidemiologic studies of cardiovascular outcomes, which have consistently linked long-term PM_2.5_ exposure to vascular and endothelial dysfunction, biological pathways that have been linked to both ED and cardiovascular disease [27]. For example, a number of studies have found significant associations between ambient PM_2.5_ and various measures of cardiovascular disease, including increased blood pressure [28–30], stroke [31], brachial artery reactivity to shear stress [15, 32], and atherosclerosis [33–35]. Similarly, PM_2.5_ has also been shown to result in endothelial dysfunction [36] and oxidative stress [37] as assessed by measurements of the associated proteins Tumor Necrosis Factor-alpha (TNF-α), intercellular adhesion molecules (iCAM), and vascular cell adhesion molecules (vCAM). In contrast to our findings, factors, such as BMI [38] and diabetes [15] have been shown to modify PM_2.5_ impacts on cardiovascular outcomes; however, our relatively small number of ED cases also likely limited our ability to detect the presence of any effect modification.

It is biologically plausible that exposure to PM_2.5_ can lead to ED. Exposure to PM_2.5_ is associated with oxidative stress and inflammation [36, 37], endothelial dysfunction [11], and atherosclerosis [33–35], potentially causing ED of vascular origin through both arteriogenic and veno-occlusive pathways [39]. Endothelial dysfunction and atherosclerosis, which are both strongly associated with ED, lead to damage in the arteries, reducing blood flow to the penis [40]. Also, in erection biology, oxidative stress and endothelial dysfunction cause a reduction in endothelial nitric oxide (NO) production, leading to an increase in smooth muscle tone, impairment of vasodilation, and impairment of the ability to achieve an erection [9, 41]. Additional support comes from animal models, where PM_2.5_ was shown to decrease NO bioavailability, which is required for penile erection [42].

Our findings that longer averages of 1 to 7 years are associated with ED are supported by studies that show higher risks for cardiovascular disease increases with longer PM_2.5_ exposure windows [43]. Implications of these findings are that adverse vascular effects of exposure to PM_2.5_ are cumulative over time, progressively leading to changes that manifest in a variety of vascular disorders, including ED. It is also interesting to note that we found lower, but still non-statistically significant associations between exposure to PM_2.5_ and prevalent cases of ED, possibly due to the fact that prevalent cases of ED may have been present for many years and are not related to the recent exposure windows used in this study.

Our study has some potential limitations that warrant discussion. First, the number of men with incident ED (132) was relatively small. The number of men with incident ED may have been underestimated in Wave 1 given that men were not asked about ED if they had no recent sexual activity, possibly due to being impotent. This potential outcome misclassification, if any, is likely small, given that multiple imputation was performed to predict incident ED status for men without ED data (39.6% in Wave 1 and 13.6% in Wave 2), with similar results to using non-imputed data. Importantly, selection bias was also unlikely, given that pollutant exposures and characteristics of men with and without ED information were similar across both data collection waves (Additional File 1: Table S1). Second, exposures were estimated using spatio-temporal models created from ambient monitor measurements that are imperfect measures of personal exposures to ambient air pollution. However, our exposure assessment method minimized exposure error resulting from spatial variation in PM_2.5_ concentrations, as the distance of the grid point estimates to residential addresses were minimal and substantially less than would otherwise occur if more traditional closest stationary ambient monitor concentrations were used to assess exposures. Any remaining exposure error would likely bias our odds ratios towards the null, as shown by previous studies that show that chronic health risks are underestimated using nearest monitor exposures [44–46]. Third, ED status was ascertained using self-reports of ED and medication use, which may result in outcome misclassification, likely biasing our results towards the null given its independence with pollutant exposures and given our binary outcome. The study would be improved by confirming ED status with clinical diagnosis. Fourth, the NSHAP study consisted of community dwelling older adults, limiting its generalizability to younger men and institutionalized men. Lastly, there are a number of confounding variables that could be included in future studies that were not available for all participants in this study, resulting in potential residual confounding. Examples include an improved measure of individual SES (beyond our use of education as an SES proxy), given its association with both higher pollutant levels [47] and ED [3], and more comprehensive information on medication usage and underlying health conditions related to ED.

Important strengths of this study include our NSHAP population, which is nationally representative of older adults. In this study, we analyzed the impacts of PM_2.5_ on incident ED controlling for key participant-specific demographic characteristics and accounting for medication use. This comprehensive characterization of each participant allowed us to control for potential confounders and to investigate the possibility of effect modification on a participant specific basis. The longitudinal nature of participant data also allowed us to capture newly developed cases of ED and analyze the impact of ambient air pollution on incident ED. Additionally, we assessed PM_2.5_ exposures for each participant using Geographic Information System (GIS) based spatio-temporal models, which reduced exposure error in our exposure estimates. Importantly, our findings were robust to our method of analysis, with results from several sensitivity analyses being qualitatively similar to those for our main analysis.

## Conclusions

In summary, we found positive associations between recent long-term ambient PM_2.5_ levels and ED that did not reach nominal statistical significance. The associations were stronger for longer-term moving averages of exposure. Given that our study is the first to examine associations between ambient air pollution and ED, further research investigating the association of PM_2.5_ and incident ED should be conducted using larger sample sizes. If the relationship between PM_2.5_ and incident ED does in fact exist, limiting exposure to PM_2.5_ may decrease the number of incident cases of ED in older men, a common condition that affects an increasing number of men.

## Additional file

Additional file 1: Erectile Dysfunction and Exposure to Ambient Air Pollution in a Nationally Representative Cohort of Older Men. **Table S1.** Wave-Specific Comparison of NSHAP Male Participants with and without Available ED Data. **Table S2.** ORs (95% CIs) for Prevalent ED per IQR Increase in PM_2.5._ **Table S3.** ORs (95% CIs) for Incident ED per IQR Increase in PM2.5: Complete Case Analysis. (DOCX 35 kb)

## Abbreviations

BMI: Body mass index
CESD: Center for epidemiological studies depression scale
CRP: C-reactive protein
ED: Erectile dysfunction
EPA: Environmental protection agency
GAMMs: Generalized additive mixed models
GIS: Geographic information systems
iCAM: Intercellular adhesion molecules
IMPROVE: Interagency monitoring of protected visual environments
IQR: Interquartile range
NO: Nitric oxide
NO_2﻿_: Nitrogen dioxide
O_3_: Ozone
OR: Odds ratio
PM_2.5_: Particulate matter with aerodynamic diameters ≤2.5 μm
SES: Socioeconomic status
TNF-α: Tumor necrosis factor-alpha
vCAM: Vascular cell adhesion molecules
