# Supplementary material for: Erectile dysfunction and exposure to ambient Air pollution in a nationally representative cohort of older Men
Source: Environ Health. 2017 Feb 17;16:12. doi: 10.1186/s12940-017-0216-6 (PMC5316194; doi:10.1186/s12940-017-0216-6)
Supplement: Additional file 1: — Erectile Dysfunction and Exposure to Ambient Air Pollution in a Nationally Representative Cohort of Older Men. Table S1. Wave-Specific Comparison of NSHAP Male Participants with and without Available ED Data. Table S2. ORs (95% CIs) for Prevalent ED per IQR Increase in PM2.5. Table S3. ORs (95% CIs) for Incident ED per IQR Increase in PM2.5: Complete Case Analysis. (DOCX 35 kb) [file 12940_2017_216_MOESM1_ESM.docx]

Additional File 1: Erectile Dysfunction and Exposure to Ambient Air Pollution in a Nationally Representative Cohort of Older Men

Lindsay A. Tallon MSPH^1,2^, Justin Manjourides PhD^1^, Vivian C. Pun PhD^1^, Murray A. Mittleman MD DrPH^3^, Marianthi-Anna Kioumourtzoglou ScD^4^, Brent Coull PhD^5^, Helen Suh ScD^6^

^1^ Department of Health Sciences, 360 Huntington Avenue, Northeastern University, Boston, MA 02115; ^2^ MCPHS University, 179 Longwood Avenue, Boston, MA 02115; ^3^ Department of Epidemiology, Harvard T.H. Chan School of Public Health, 677 Huntington Ave., Boston, MA 02115; ^4^ Department of Environmental Health Sciences, Columbia University Mailman School of Public Health, 722 W. 168th Street, #1105C, New York NY 10032; ^5^ Department of Biostatistics, Harvard T.H. Chan School of Public Health, 677 Huntington Ave., Boston, MA 02115; ^6^ Department of Civil and Environmental Engineering, Tufts University, 200 College Avenue, Medford, MA

**Table of Contents**

Table S1. Wave-Specific Comparison of NSHAP Male Participants with and without Available ED Data

Table S2. ORs (95% CIs) for Prevalent ED per IQR Increase in PM_2.5_

Table S3. ORs (95% CIs) for Incident ED per IQR Increase in PM2.5: Complete Case Analysis

**Table S1.** Wave-Specific Comparison of NSHAP Male Participants with and without Available ED Data ^a^

| **Characteristic** | **Wave 1**  **With ED Data** | **Wave 1**  **No ED Data** | **Wave 2**  **With ED Data** | **Wave 2**  **No ED Data** |
| --- | --- | --- | --- | --- |
| **Number of Participants** | 878 | 576 | 1327 | 211 |
| **Age**, years (SD) | 66.37 (6.86) | 72.20 (7.59)* | 72.69 (7.43) | 75.34 (7.68)* |
| **Race**, n (%)  Non-Hispanic white  Non-Hispanic black  Hispanic non-black  Other | 628 (71.77%)  126 (14.40%)  96 (10.97%)  25 (2.86%) | 403 (70.09%)  98 (17.04%)  57 (9.91%)  17 (2.96%) | 949 (71.93%)  181 (13.68%)  157 (11.87%)  36 (2.72%) | 155 (73.46%)  36 (17.06%)  17 (8.06%)  3 (1.42%) |
| **Education Level**, n (%)  Less than High School  H.S. or vocational school  College degree or greater | 157 (17.88%)  450 (51.25%)  271 (30.87%) | 162 (28.13%)*  282 (48.96%)*  132 (22.92%)* | 261 (19.67%)  661 (49.81%)  405 (30.52%) | 43 (20.38%)*  119 (56.40%)*  49 (23.22%)* |
| **Diabetes**, n (%) | 172 (19.59%) | 159 (27.60%)* | 334 (25.28%) | 58 (27.75%) |
| **Hypertension ^b^**, n (%) | 407 (47.33%) | 260 (46.18%) | 599 (46.54%) | 89 (44.95%) |
| **Elevated CRP,** (>1), n (%) | 301 (52.35%) | 228 (64.23%)* | 848 (70.73%) | 131 (73.60%) |
| **BMI** (kg/m^2^)  Obesity ^c^, n (%) | 29.27 (5.54)  324 (38.99%) | 28.35 (5.47)*  180 (34.35%)* | 29.39 (5.55)  496 (39.37%) | 28.70 (5.69)  63 (32.64%)* |
| **Exercise ^d^**, n (%) | 758 (86.33%) | 433 (75.17%)* | 803 (60.51%) | 116 (54.98%) |
| **Current smoker**, n (%) | 137 (15.64%) | 101 (17.53%) | 210 (15.83%) | 33 (15.64%) |
| **Elevated depression** ^e^, n (%) | 120 (13.70%) | 173 (30.03%)* | 237 (17.86%) | 42 (19.91%) |

^a^ Those with data answered ED question or responded yes to medication, 1454 total males in wave 1, 1538 total in wave 2, asterisks indicate p <0.05 for difference between groups using chi-square tests for categorical variables and t-test for difference in means

^b^ >90 diastolic and/or >140 systolic averaged from 2 or 3 blood pressure measurements

^c^ BMI >30 kg/m^2^

^d^ Exercise defined as 1 or more times/week

^e^ Elevated depression defined as a score of 9 or greater on the CESD-11

^*^ p < 0.05

**Table S2.** ORs (95% CIs) for Prevalent ED per IQR Increase in PM_2.5_ ^a^

|  | **PM_2.5_ Moving Average** ^b^ | | | | | | |
| --- | --- | --- | --- | --- | --- | --- | --- |
|  | **1 year** | **2 year** | **3 year** | **4 year** | **5 year** | **6 year** | **7 year** |
| **Base Model** ^c^ | 0.96  (0.76, 1.22) | 0.96  (0.77, 1.19) | 0.96  (0.78, 1.18) | 0.97  (0.80, 1.19) | 0.98  (0.81, 1.19) | 0.98  (0.81, 1.19) | 0.98  (0.81, 1.18) |
| **Adjusted Model** | 1.06  (0.81, 1.37) | 1.06  (0.83, 1.36) | 1.06  (0.83, 1.34) | 1.06  (0.85, 1.33) | 1.06  (0.85, 1.32) | 1.06  (0.85, 1.31) | 1.06  (0.86, 1.30) |

^a^ Prevalent ED is defined as either replying yes to ED question or reporting ED medication usage in either wave (n=746); results given are ORs (95% Confidence Intervals);

^b^ PM_2.5_ 1yr IQR=3.25 µg/m^3^, 2yr IQR =3.12 µg/m^3^, 3 yr IQR=3.18 µg/m^3^, 4 yr IQR = 3.12 µg/m^3^, 5yr IQR =3.10 µg/m^3^, 6 yr IQR=3.15 µg/m^3^, 7 yr IQR = 3.10 µg/m^3^

^c^ Logistic regression models include adjustment for age and geographic region (West, Midwest, Central, South, Northeast) in base models

^d^ Additional adjustment for ethnic group, education (<H.S., H.S., College), current smoking status, obesity, diabetes, depression, season, and median household income in adjusted models

**Table S3.** ORs (95% CIs) for Incident ED per IQR Increase in PM2.5: Complete Case Analysis ^a^

|  | **PM_2.5_ Moving Average** ^b^ | | | | | | | | | | | | |
| --- | --- | --- | --- | --- | --- | --- | --- | --- | --- | --- | --- | --- | --- |
|  | **1 year** | **2 year** | | **3 year** | | **4 year** | | **5 year** | | **6 year** | | **7 year** | |
| **Base Model** ^c^ (n=400) | 1.03  (0.74, 1.45) | | 1.02  (0.74, 1.41) | | 1.03  (0.76, 1.41) | | 1.05  (0.79, 1.41) | | 1.06  (0.80, 1.40) | | 1.05  (0.80, 1.39) | | 1.05  (0.80, 1.38) |
| **Adjusted Model** ^d^  (n=400) | 1.11  (0.76, 1.61) | | 1.13  (0.79, 1.61) | | 1.14  (0.81, 1.61) | | 1.15  (0.83, 1.59) | | 1.14  (0.84, 1.57) | | 1.14  (0.84, 1.56) | | 1.14  (0.84, 1.54) |

^a^ Incident ED is defined as either replying yes to ED question or reporting ED medication use in Wave 2 but not in Wave 1; 130 men with data available had incident ED compared to 270 men without ED in either wave; results given are ORs (95% Confidence Intervals);

^b^ PM_2.5_ 1yr IQR=3.25 µg/m^3^, 2yr IQR =3.12 µg/m^3^, 3 yr IQR=3.18 µg/m^3^, 4 yr IQR = 3.12 µg/m^3^, 5yr IQR =3.10 µg/m^3^, 6 yr IQR=3.15 µg/m^3^, 7 yr IQR = 3.10 µg/m

^c^ Logistic regression models include adjustment for age and geographic region (West, Midwest, Central, South, Northeast) in base models

^d^ Additional adjustment for ethnic group, education (<H.S., H.S., College), current smoking status, obesity, diabetes, depression season, and median household income in adjusted models
